# Position-5–driven reorientation of an immunodominant HLA-A*24:02 SARS-CoV-2 epitope drives universal T cell escape

**DOI:** 10.1172/jci.insight.202235

**Published:** 2026-03-17

**Authors:** Takeshi Nakama, Aaron Wall, Garry Dolton, Li Rong Tan, Hannah Thomas, Hiroshi Hamana, Yoshiki Aritsu, Toong Seng Tan, Mako Toyoda, Yoshihiko Goto, Huanyu Li, Mizuki Kitamatsu, Keiko Udaka, Yusuke Miyashita, Hiroyuki Oshiumi, Kimitoshi Nakamura, Yoji Nagasaki, Rumi Minami, Hirotomo Nakata, Pierre J. Rizkallah, Hiroyuki Kishi, Takamasa Ueno, Andrew K. Sewell, Chihiro Motozono

**Affiliations:** 1 Division of Infection and Immunity, Joint Research Center for Human Retrovirus infection, Kumamoto University, Kumamoto, Japan.; 2 Division of Infection and Immunity, Cardiff University School of Medicine, Cardiff, Wales, United Kingdom.; 3 Department of Immunology, Faculty of Medicine, Academic Assembly, University of Toyama, Toyama, Japan.; 4 Department of Applied Chemistry, Faculty of Science and Engineering, Kindai University, Osaka, Japan.; 5 Department of Immunology, Kochi University, Kochi, Japan.; 6 Department of Immunology, Graduate School of Medical Sciences, Faculty of Life Sciences, and; 7 Department of Pediatrics, Graduate School of Medical Sciences, Kumamoto University, Kumamoto, Japan.; 8 Division of Infectious Diseases, Clinical Research Institute, NHO, Kyushu Medical Center, Fukuoka, Japan.; 9 Internal Medicine, Clinical Research Institute, NHO, Kyushu Medical Center, Fukuoka, Japan.; 10 Department of Hematology, Rheumatology and Infectious Diseases, Kumamoto University School of Medicine, Kumamoto University Hospital, Kumamoto, Japan.

**Keywords:** Immunology, Infectious disease, COVID-19, T cell receptor, T cells

## Abstract

Cytotoxic T lymphocytes form a critical component of SARS-CoV-2 immunity by recognizing viral peptides bound to HLA class I molecules. Here, we identified the spike-derived peptide NYNYLYRLF_448-456_ (NF9) as the immunodominant HLA-A*24:02–restricted epitope in both convalescent and vaccinated donors. Across cohorts, A24/NF9-specific responses were dominated by public TCR motifs featuring TRAV12-1 paired with TRBJ2-7 and a conserved CDR3β sequence (CASSXXXGYEQYF). Using a panel of 13 TCRs, we mapped recognition of single amino acid substitutions within NF9 and identified residue 5 (L452) as the principal determinant of escape. The L452R substitution, characteristic of the Delta variant, abolished recognition across all tested TCRs despite preserved HLA binding. Crystallography of a representative public TCR (P1-15) revealed that mutation at position-5 reoriented the peptide within HLA-A*24:02, flipping the adjacent Y453 side chain into the peptide-binding groove and eliminating the dominant TCR contact. This position-5–driven conformational switch provided a structural mechanism for universal loss of NF9 recognition by HLA-A*24:02–restricted T cells. Consistent with this, Delta-infected convalescents failed to mount de novo NF9-5R–specific responses while retaining responses to the conserved A24/QI9 spike epitope. Together, these findings defined the basis of A24/NF9 recognition and showed how 1 mutation remodeled peptide presentation to abrogate TCR responses.

## Abstract 2

A mutation in SARS-CoV-2 flips a viral fragment, hiding it from immune T-cells and allowing the virus to evade an important immune response.

## Graphical abstract

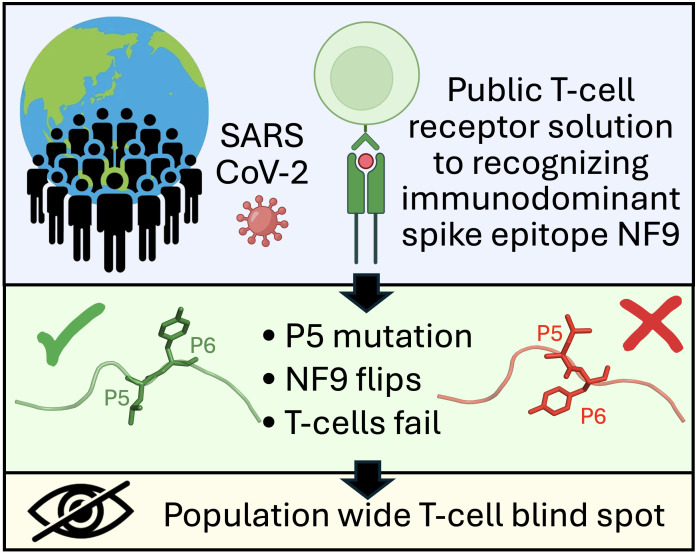

## Introduction

Cytotoxic T lymphocytes (CTLs) play a central role in antiviral immunity by detecting and eliminating infected cells that present viral peptides on human leukocyte antigen (HLA) class I molecules. In SARS-CoV-2 infection, CTLs play a pivotal role in immune protection and are associated with reduced disease severity (1, 2). Indeed, individuals can control infection in the absence of detectable antibodies, indicating that cellular immunity alone can confer protection (3–5). Robust CTL responses against conserved coronavirus epitopes are linked to milder COVID-19 (6), and the HLA-B*15:01 allele has been associated with asymptomatic infection, likely due to preexisting, cross-reactive T cells primed by other β-coronaviruses (7).

Given the selective pressure imposed by CTLs, and drawing from experience with chronic viral infections like HIV-1 (8), it was anticipated that SARS-CoV-2 would evolve mutations conferring escape from T cell recognition as the COVID-19 pandemic progressed (9). Intrahost longitudinal analyses have confirmed that escape from CD8^+^ T cells contributes substantially to SARS-CoV-2 evolution (10), and several reports have shown that escape variants can be transmitted and propagated (9, 11, 12). We previously demonstrated that the spike L452R mutation, which emerged in the Delta variant, enabled escape from HLA-A*24:02–restricted NF9-specific CTLs in both convalescent and vaccinated individuals (13, 14). Likewise, we reported that the P272L mutation in the spike protein that circulated during the second wave of the COVID-19 pandemic escaped from HLA-A*02:01–restricted CTLs elicited by infection or vaccination (9). Escape from T cell recognition is not confined to CD8^+^ cells but extends to CD4^+^ responses (15, 16). While the diversity of HLA alleles across the population makes complete T cell escape by SARS-CoV-2 improbable (17), dominant responses restricted by common HLAs are expected to leave mutational “footprints” in the viral genome as seen in HIV-1 (18–21). Tracking of the H3N2 influenza virus since the 1968 Hong Kong pandemic indicates that similar immune-driven footprints have resulted in the loss of a predominant CD8 T cell epitope approximately every 3 years (22). Understanding how viral mutations subvert T cell immunity is crucial for vaccine design, predicting variant evolution, and strengthening preparedness for future pandemics.

Against this backdrop, we focused on CTL responses restricted by HLA-A*24:02, a globally prevalent HLA allele enriched in East Asia and several Indigenous populations across Oceania and the Americas, and present in ~60% of the Japanese cohort we studied (23). The prominence of the HLA-A*24:02 allele in regions housing over half of the world’s population, makes it particularly relevant to global resistance to emerging infectious diseases. HLA-A*24:02 is of further interest in SARS-CoV-2 infection as it is associated with a decreased risk of severe outcomes (23). Some CTL responses, especially those that target constrained regions of viral proteins, are likely to present more of a challenge for viruses to escape from. Indeed, immunodominant CTL have been linked to control of HIV-1 viremia (8, 24) and public cross-reactive T cell receptors (TCRs) can mediate such control in an HLA-dependent manner (24–26). Consequently, monitoring of SARS-CoV-2 mutation in the context of dominant T cell responses offers valuable insight for rational vaccine design and may inform strategies against future emerging pathogens.

Here, we define the molecular basis of immune recognition and escape at the immunodominant HLA-A*24:02–restricted SARS-CoV-2 spike_448-456_ epitope NYNYLYRLF (NF9). We previously reported that immune escape at this epitope often involves substitution of the leucine at position-5 (P5), suggesting its importance for TCR engagement (13, 14). However, structural and biophysical analyses reveal that the adjacent tyrosine at position 6 (P6) forms the dominant TCR contact, accounting for over a quarter of peptide-TCR interactions. Surprisingly, the P5 L452R mutation induces a conformational rearrangement in A24/NF9 that reorients the P6 tyrosine toward the HLA cleft, rendering it inaccessible to TCRs and thereby enabling population-wide immune escape.

## Results

### NF9 is the dominant spike epitope in HLA-A*24:02^+^ vaccinees.

**Figure 1:**
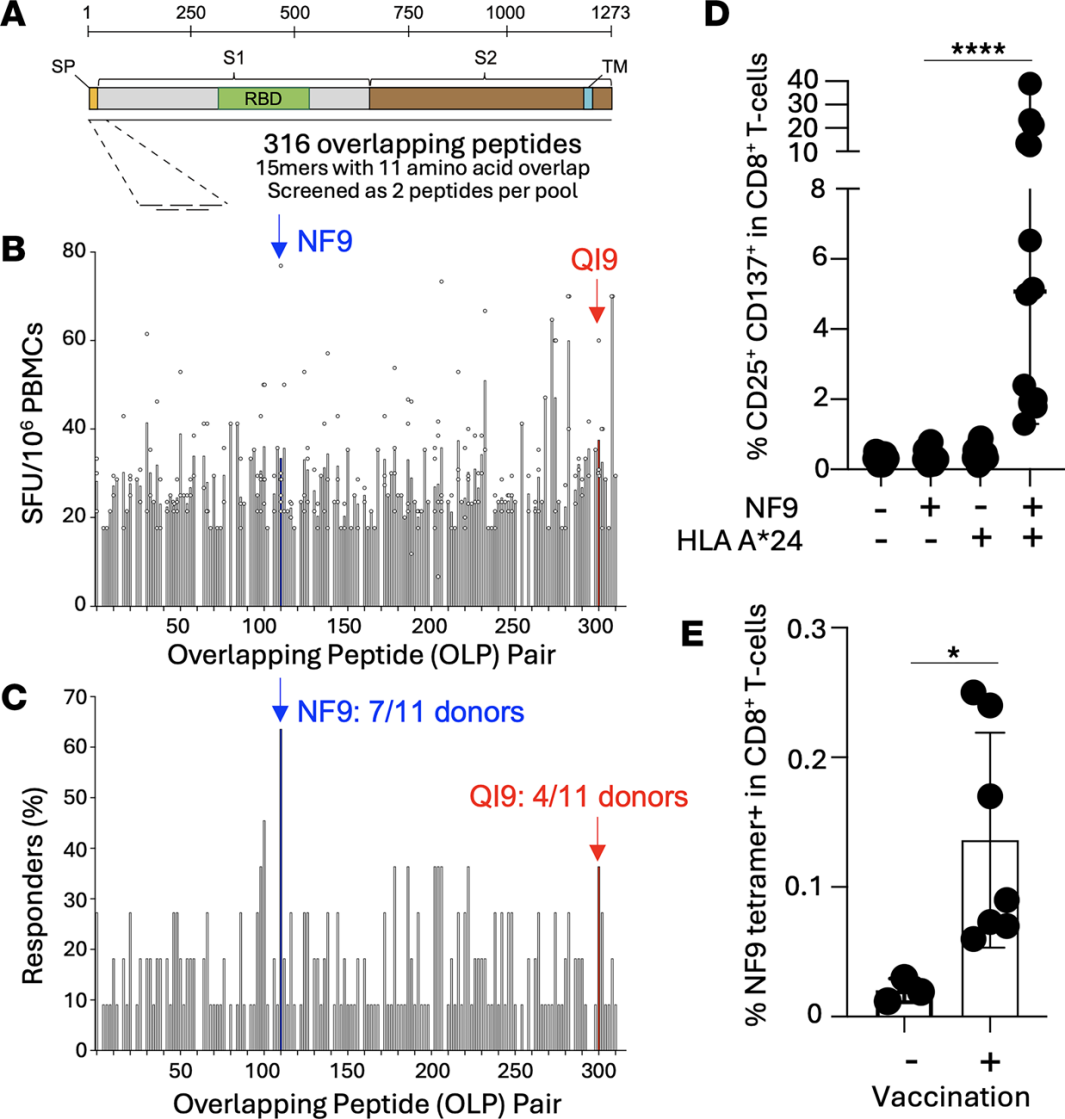
Immunodominant NF9/A24-specific T cell responses in vaccinated donors. (**A**) Scaled schematic of SARS-CoV-2 spike the S1 and S2 subunits, signal peptide (SP), receptor binding domain (RBD), and transmembrane domain (TM). Overlapping peptides spanning the spike peptide used for functional assays. (**B** and **C**) Magnitude of T cell responses, and fraction of HLA-A*24:02^+^ donors responding to overlapping peptide (OLP) pairs spanning SARS-CoV-2 spike protein (*n* = 11) as measured by IFN-γ ELISpot. Each well contained 2 overlapping peptides (11-aa overlap). Positive responses were defined as > 3 spot-forming cells above background. OLP pairs 111–112 and 301–302, which encompasses the A24/NF9 and the A24/QI9 epitopes, respectively, are indicated in red and blue with color-matched arrows. (**D**) CD25^+^CD137^+^CD8^+^ T cells from HLA-A*24:02^+^ and HLA-A*24:02^–^ donors following 14-day incubation ± NF9 peptide. Summary data from all donors (*n* = 14) (****P < 0.0001 by Mann-Whitney *U* test ± NF9 peptide is indicated). Gating strategy is shown in Supplemental Figure 1A. (**E**) Detection of A24/NF9-specific T cells in PBMCs using A24/NF9 tetramers in vaccinated and unvaccinated HLA-A*24:02^+^ donors (*P = 0.0167, Mann-Whitney *U* test). Representative plots are shown in Supplemental Figure 1B.

The HLA-A*24:02 allele is the most prevalent HLA class I allele in Southeast Asian populations and one of the most widely distributed globally (27). During the COVID-19 pandemic, an inverse correlation was observed between SARS-CoV-2–related deaths per 100,000 population and the prevalence of HLA-A*24:02, highlighting the potential protective role of this allele (23). We and others previously identified 2 immunodominant SARS-CoV-2 spike–derived epitopes presented by HLA-A*24:02: NYNYLYRLF (A24/NF9, residues 448–456) and QYIKWPWYI (A24/QI9, residues 1208–1216) (13, 14, 28, 29). To comprehensively assess the relative immunodominance of these epitopes within the broader spike-specific T cell response, we screened PBMCs from 11 HLA-A*24:02*^+^* individuals who had received 2 doses of BNT162b2 or mRNA-1273. IFN-γ ELISpot assays were performed with overlapping 15-mer peptides (11–amino acid overlap) spanning the entire spike protein (method as in ref. 30; donor details in Supplemental Table 1; supplemental material available online with this article; https://doi.org/10.1172/jci.insight.202235DS1) (Figure 1A). To maximize the use of available PBMCs, 2 overlapping peptides (OLPs) were screened per well, allowing coverage of the full spike sequence with limited cell numbers. Strong ex vivo T cell responses were detected against OLP pairs 111–112 and 301–302, which incorporate the NF9 and QI9 epitopes, respectively. Among all peptide pairs tested, the NF9-containing peptide (OLP112) elicited the most robust (Figure 1B) and frequent (Figure 1C) responses, confirming A24/NF9 as the dominant HLA-A*24:02–restricted spike epitope in mRNA vaccinees. Responses to A24/NF9 were observed in 7 of 11 donors, while A24/QI9 responses were detected in 4 of 11 (Figure 1C), consistent with previous tetramer-based studies (14). To validate this immunodominance, PBMCs from HLA-A*24:02*^+^* (*n* = 14) and HLA-A*24:02^neg^ (*n* = 13) vaccinated donors were stimulated with the NF9 peptide and cultured for 14 days. T cell activation, measured by coexpression of CD25 and CD137 revealed that NF9-specific CD8*^+^* T cells were induced in all HLA-A*24:02^+^ individuals (median: 5.15%) but not in HLA-A*24:02^neg^ donors (median: 0.30%) (***P < 0.0001, Figure 1D and Supplemental Figure 1, A and B), confirming HLA-A*24:02 restriction. Direct ex vivo tetramer staining further supported this conclusion. Vaccinated HLA-A*24:02*^+^* donors had significantly higher frequencies of A24/NF9 tetramer^+^ CD8*^+^* T cells compared with unvaccinated HLA-A*24:02^+^ controls (*P* = 0.0167; Figure 1E and Supplemental Figure 1, C and D). Moreover, across 9 vaccinated HLA-A*24:02^+^ donors, the frequency of A24/NF9 tetramer^+^ cells strongly correlated with the magnitude of NF9-specific CD8^+^ T cell responses after in vitro peptide expansion (*R* = 0.9006, ***P = 0.0009; Supplemental Figure 1E). Together, these data establish A24/NF9 as the dominant spike-derived epitope in HLA-A*24:02^+^ vaccinees and confirm that the response is strictly dependent on HLA-A*24:02 for presentation. Next, we analyzed A24/NF9 and A24/QI9 specific T cells in both vaccinated and convalescent donors.

### The response to HLA-A*24:02 NF9 involves shared TCR features across donors.

**Figure 2:**
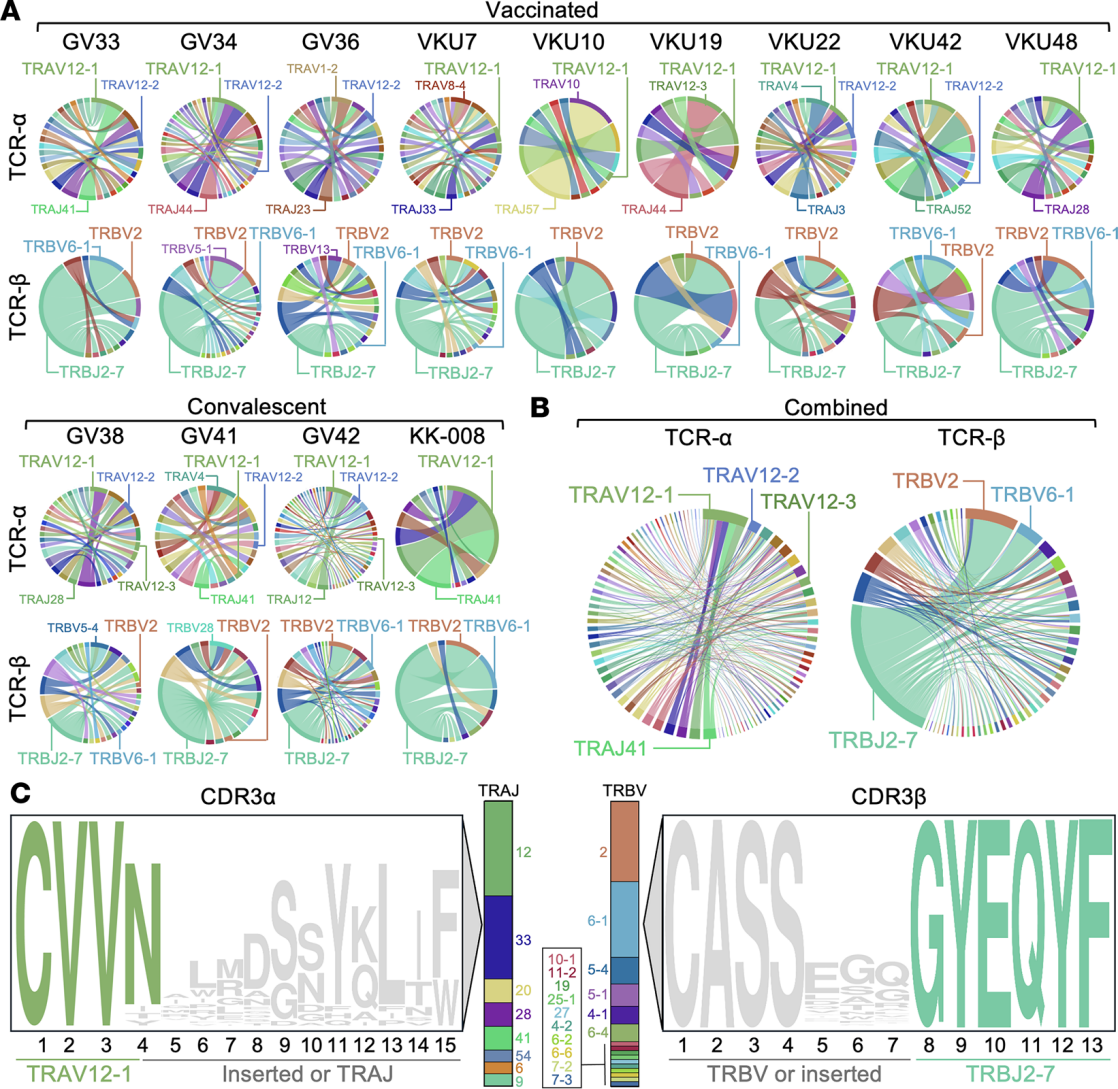
T cell receptor analysis of NF9/A24 and QI9/A24-specific T cells. (**A**) TRAV–TRAJ and TRBV–TRBJ gene usage among NF9-specific T cells from 9 vaccinated and 4 convalescent HLA-A*24:02^+^ donors. Circos plots depict relative usage of TRAJ/TRBJ (left arcs) and TRAV/TRBV (right arcs), with arc size reflecting gene frequency. Ribbons indicate V–J pairings. (**B**) Combined Circos plots from all 13 donors (vaccinated + convalescent) showing TCRα and TCRβ chain usage. A strong bias toward TRAV12-1 and TRBJ2-7 is evident in the α and β chains, respectively. (**C**) Sequence logo plots showing CDR3α (left) and CDR3β (right) motifs derived from the combined donor set. CDR3α: TRAV12-1^+^ sequences, most common length = 15 aa. CDR3β: TRBJ2-7^+^ sequences, most common length = 13 aa. Colored bars at the center indicate the diversity of TRAJ (α chain) and TRBV (β chain) gene usage contributing to each motif, ordered by frequency. See Supplemental Figures 2–4 for additional TCRα/β characteristics, including full CDR3 length distributions.

Single-cell sorting of ex vivo PBMCs was performed following A24/NF9 tetramer staining (31) (Supplemental Tables 1 and 2), and the TCR repertoires of A24/NF9 specific T cells were analyzed for vaccinated (*n* = 9) and convalescent donors (*n* = 4). The TCRα and TCRβ sequences revealed clear convergence in repertoire usage. Most notably, we observed recurrent use of the *TRBJ2-7* gene segment (CDR3 amino acid residues GYEQYF) for both vaccinees and convalescent donor (Figure 2, A and B, and Supplemental Figures 2–4), which contributed to a highly conserved CDR3β motif in the form CASSXXXGYEQYF, where “X” represents variable residues (Figure 2C and Supplemental Figures 2–4). This public CDR3β motif was frequently associated with TRBV2 or TRBV6-1 (contributing CDR3 amino acid residues CASS) (Figure 2B and Supplemental Figures 2–4). For the TCRα chains, the *TRAJ* gene segments usage was highly varied, and for TRAV genes, there was a bias toward *TRAV12-1* (Figure 2, A–C, and Supplemental Figures 2–4). The CDR3β motif and public TRAV12-1^+^ clonotypes were also detected in A24/NF9-specific CD8^+^ T cells from other published cohorts (32, 33) (Supplemental Figure 5). These data demonstrate that this TCR pattern is consistent across studies and independent of whether T cells were induced by vaccination or infection, indicating a strong structural selection for TCRs that can productively engage the A24/NF9 complex.

### TCR cross-reactivity at P5 of the HLA-A*24:02 NF9 peptide.

**Figure 3:**
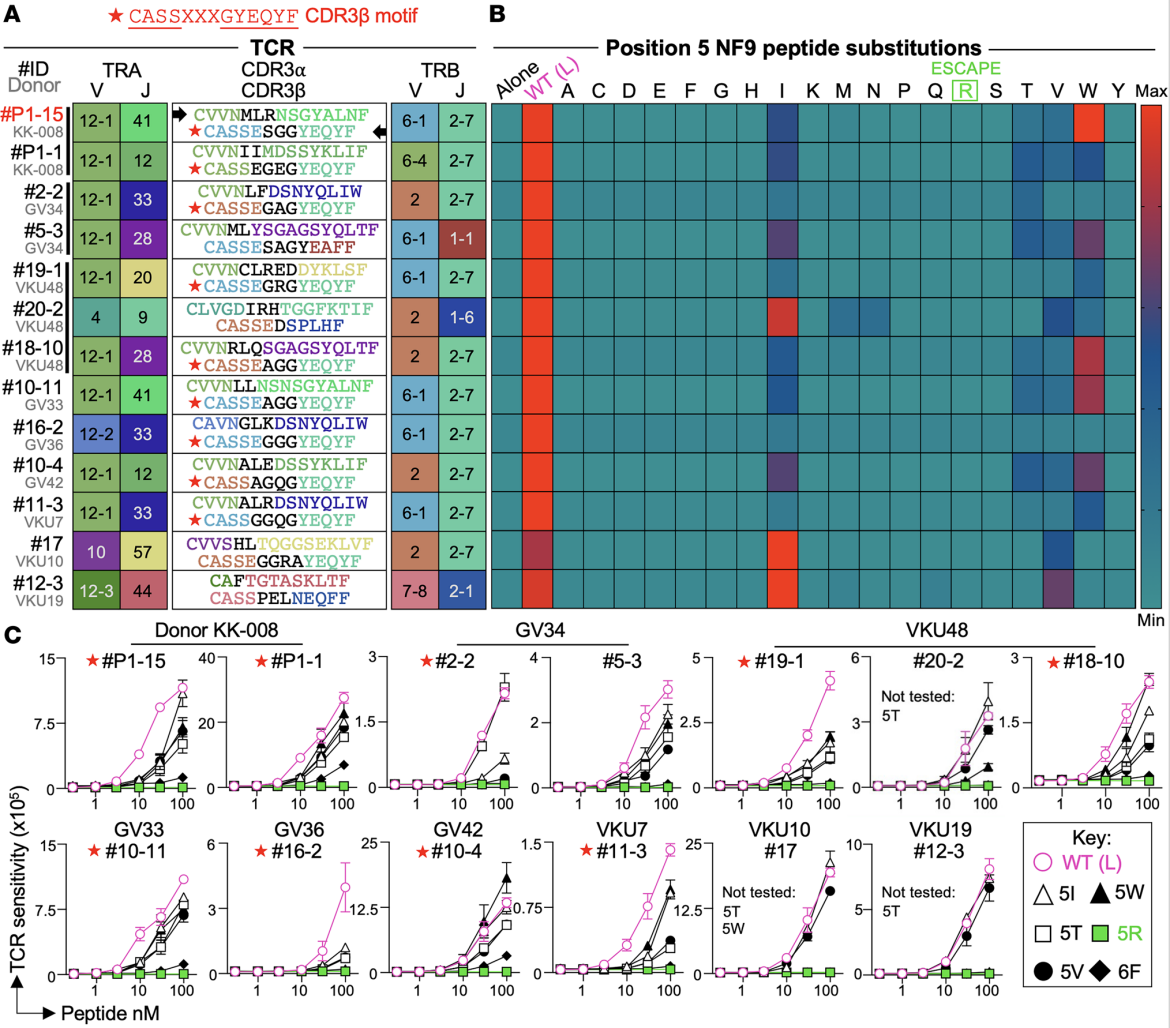
Preference for leucine and nonrecognition of arginine at P5 by NF9-specific TCRs. (**A**) Paired TCRs from vaccinated or convalescent HLA-A*24:02^+^ donors that recognize the NYNYLYRLF_448-456_ spike peptide. Each TCR’s variable (V) and joining (J) segments and corresponding CDR3 sequences are shown. V and J segments are color-coded to match the Circos plots used elsewhere in the study. Black text in CDR3 sequence indicates amino acids derived from P or N nucleotide additions or, for TRB chains, from diversity (D) segments. TRAV12-1 expression and the conserved CDR3β motif CASSXXXGYEQYF (red star) identify canonical NF9-specific TCRs, with other clonotypes included for comparison. (**B**) Heatmap summarizing Jurkat reporter cell activation for each TCR in **A** against single amino acid substitutions at position-5 of the NF9 peptide. Responses are normalized to each TCR’s maximal activation to enable parallel comparison. (**C**) Titration assays of each TCR from **A** expressed in Jurkat cells, tested against the position-5 variants recognized in **B**, as well as the 5R (L452R) escape mutant and the 6F (Y453F) variant identified in farmed mink (37).

The L452 residue of the SARS-CoV-2 spike protein lies within the receptor-binding domain and forms the fifth position (P5) of the A24/NF9 epitope. Mutations at this site, including L452R and L452Q, were characteristic of the Delta, Epsilon, and Lambda variants and later appeared in multiple Omicron-derived lineages such as BA.5, BQ.1 BA.2.86 and JN.1 (34, 35). To define how amino acid substitutions at this position affect immune recognition, we synthesized a panel of NF9/5X peptides (a positional “X-scan” where “X” represents any proteogenic amino acid substitution at P5). All variant peptides bound efficiently to HLA-A*24:02 (Supplemental Table 3), consistent with previous report that this position is not a primary HLA anchor (36). We next evaluated TCR recognition using 8 representative A24/NF9 TCRs using TRAV12-1 and incorporating the CDR3β motif CASSXXXGYEQYF (TRBV2 or TRBV6-1, and TRBJ2-7), and additionally 5 other TCRs with similar features to that of the canonical TCRs (Figure 3A and Supplemental Figure 6 for NF9 tetramer staining). Across all 13 TCRs, the index leucine at P5 was the most potent agonist (Figures 3, B and C). Fifteen of 20 substitutions abolished activation across the TCRs, including the naturally occurring L452R, L452Q, and L452M mutations (Figure 3B). Only isoleucine, threonine, valine, and tryptophan were tolerated, and even these substitutions produced weaker responses (Figure 3, B and C). Peptide titrations confirmed that NF9-5L from the Wuhan strain was the strongest agonist, while NF9-5R was not recognized by any TCR even at 100 nM peptide (Figure 3C). For completeness, we also included the Y453F mutation (NF9-6F) associated with a SARS-CoV-2 outbreak in farmed Mink (37) (Figure 3C). These data indicate that TCRs using TRAV12-1—and possessing the CDR3β CASSXXXGYEQYF motif—as well as noncanonical TCRs have limited capacity to tolerate amino acid substitutions at P5 of the NF9 peptide. These data confirm that the NF9-5L sequence found in the original SARS-CoV-2 Wuhan strain and present in COVID-19 vaccines was the strongest agonist for these T cells. Importantly, none of the NF9-specific T cell clones, including those with non-canonical TCR pairings, responded to NF9-5R. Together these findings demonstrate that all NF9-specific clonotypes, show a strong preference for the NF9-5L Wuhan sequence. The L452R substitution found in Delta and subsequent variants therefore provides profound, population-wide escape from A24/NF9-specific T cell recognition. We next explored this escape further by examining the binding of a canonical TCR to NF9 variants.

### Strong P1-15 TCR binding to HLA-A*24:02-NF9 but weak or absent binding to all other NF9 variants.

**Figure 4:**
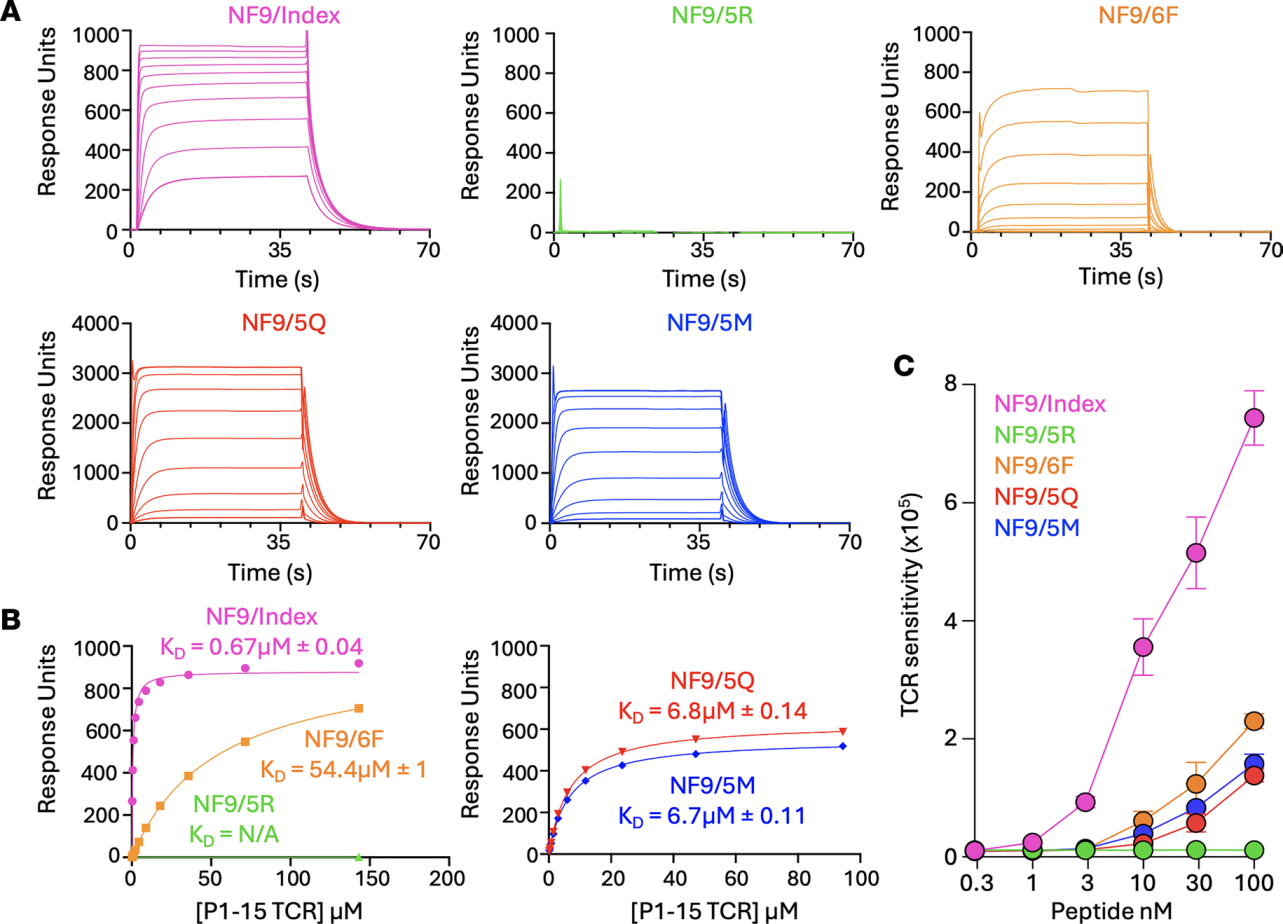
Binding affinity and cross-reactivity of P1-15:A24/NF9 interaction. (**A**) Surface plasmon resonance (SPR) analysis of P1-15 TCR binding to HLA-A*24:02 complexes presenting NF9 (magenta), NF9-6F (orange), NF9-5R (green), NF9-5Q (red), and NF9-5M (blue). (**B**) SPR responses to 10 serial dilutions of P1-15 TCR were measured. K_D_s were determined by non-linear fit curve (y = [P1 x]/[P2 + x]). (**C**) NFAT-luciferase reporter activity in Jurkat cells transduced with P1-15 TCR and stimulated with NF9 or variant peptides (NF9-5R, NF9-6F, NF9-5Q, and NF9-5M), demonstrating functional sensitivity corresponding to the measured binding affinities.

To investigate the molecular basis of A24/NF9 recognition and cross-reactivity, we focused on representative public TCR P1-15, isolated from convalescent donor KK-008 (TRAV12-1, TRBV6-1, CDR3β CASSSGGGYEQYF, and TRBJ2-7). Surface plasmon resonance (SPR) was used to quantify equilibrium binding affinities between the P1-15 TCR and HLA-A*24:02 complexes presenting either the wild-type or variant NF9 peptides (Figure 4A). Most antiviral TCR-pHLA class I interactions exhibit affinities in the range of K_D_ = 1–10 μM (38). In contrast the P1-15 TCR bound to A24/NF9 with a KD of 0.67 μM, placing it among the highest affinity natural antiviral TCRs reported to date and suggesting that this interaction is close to the upper limit for naturally occurring A24/NF9-specific TCRs. This unusually strong binding is compatible with, but does not itself define, the convergent public architecture observed among A24/NF9-specific clonotypes.

Despite this high affinity, P1-15 exhibited no detectable binding to A24/NF9-5R and only weaker affinity for NF9-5Q (K_D_ = 6.8 μM), NF9-5M (K_D_ = 6.7 μM) and NF9-6F (K_D_ = 54.4 μM) (Figure 4B). These affinities closely mirrored functional responses measured in T cell activation assays (Figure 4C). Deng et al. reported affinities for 2 similar TRAV12-1/TRBV6-1 NF9-specific TCRs, NYN-I and NYN-II, were 13.6 and 8.6 μM (39), more than 10 times weaker than P1-15 yet still within the expected antiviral range. Given its high affinity and robust expression yield, we selected P1-15 for crystallization with A24/NF9 and its variant complexes to define the structural basis for immune escape.

### P1-15 TCR recognition is governed by NF9 peptide conformation within HLA-A*24:02.

**Figure 5:**
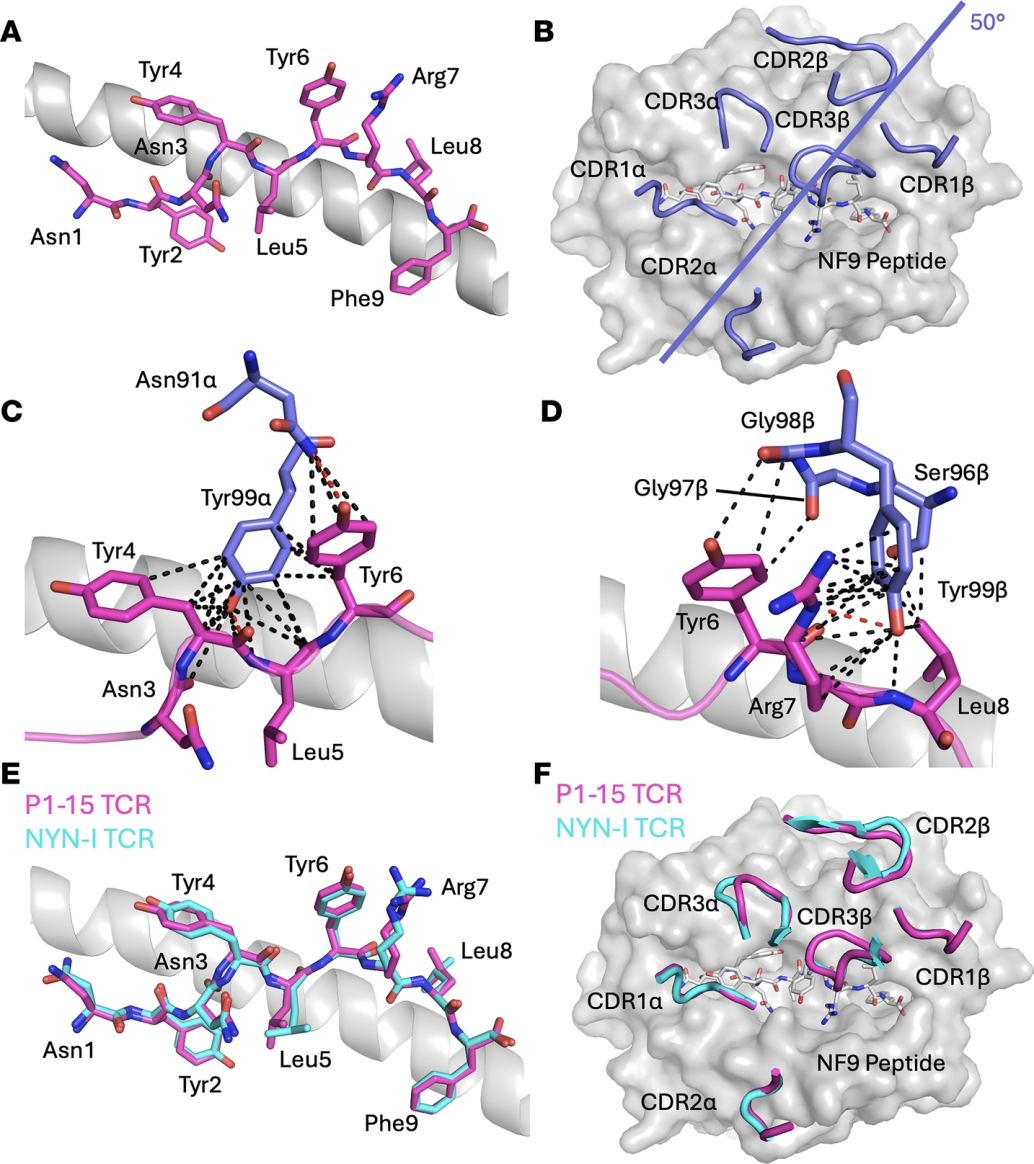
Structural analysis of the public P1-15 TCR/NF9-A24 complex. (**A**) Presentation of the NF9 peptide (magenta sticks) when in complex with the P1-15 TCR. (**B**) Top-down view of the NF9 peptide (white sticks) showing the distribution of the P1-15 CDR loops (blue cartoon). The crossing angle is indicated by the blue line. (**C** and **D**) Detailed views of contacts between CDR1α (**C**), CDR3β (**D**), and CDR3α and the NF9 peptide (magenta sticks) showing Van der Waals (black dotted lines) and hydrogen (red dotted lines) interactions. (**E**) Structural comparison of the NF9 peptide (sticks) bound to P1-15 TCR (magenta) or NYN-I TCR (cyan). (**F**) Top-down comparison of the P1-15 (blue cartoon) and NYN-I (cyan cartoon) CDR loop distributions over the NF9 peptide (white sticks).

To elucidate the structural basis for P1-15 TCR recognition, we crystallized P1-15 in complex with A24/NF9 and solved the structure at 3.1 Å resolution (Supplemental Figure 7 and Supplemental Table 4). All structures described in this study have been deposited and validated in the Protein Data Bank (P1-15:HLA-A*24:02–NF9 PDB: 28IL, HLA-A*24:02–NF9-6F PDB: 8RJH, HLA-A*24:02–NF9-5R PDB: 8RJI). Note that the P1-15 TCR β chain construct contained a short N-terminal extension resulting from the expression system, and the PDB numbering therefore begins from the first residue of this construct. For consistency with the published literature (e.g., PDB: 8YE4), amino acid numbering in this manuscript follows the canonical TCR sequence — i.e., –3 relative to the PDB files for the β chain. In the P1-15/A24/NF9 complex residues Tyr4, Tyr6, and Arg7 of the peptide projected upward toward the TCR, whereas Tyr2 and Phe9 were buried as canonical HLA-A*24:02 anchor residues (Figure 5A). Analysis of the molecular contacts between the P1-15 TCR and the NF9 peptide (Supplemental Table 5) revealed that the TCR interacted extensively through CDR1α (23% of TCR contacts), CDR3α (37%), and CDR3β (40%) (Figure 5B). Tyr4, Tyr6, and Arg7 of the peptide were key contact residues for the CDR3α and CDR3β (Figure 5, C and D). The CDR3β dominance reflected a conserved Tyr99β residue that contributed 18 van der Waals and 1 hydrogen bonds in P1-15 TCR recognition with NF9-7R and NF9-8L, explaining the recurrent TRBJ2-7 encoded CDR3β motif (CASSXXXGY99EQYF, TRBJ2-7 amino acids underlined) across different donors. The recently published A24/NF9–NYN-I TCR complex (39) (bearing similar TRAV12-1/TRBV6-1 pairing, TRBJ2-7 usage and CDR3β motif) bound NF9 in the same “P5-down, P6-up” configuration and with similar TCR CDR3 loop distribution (Figure 5, E and F), indicating that public NF9-specific TCRs converge on a shared structural solution.

**Figure 6:**
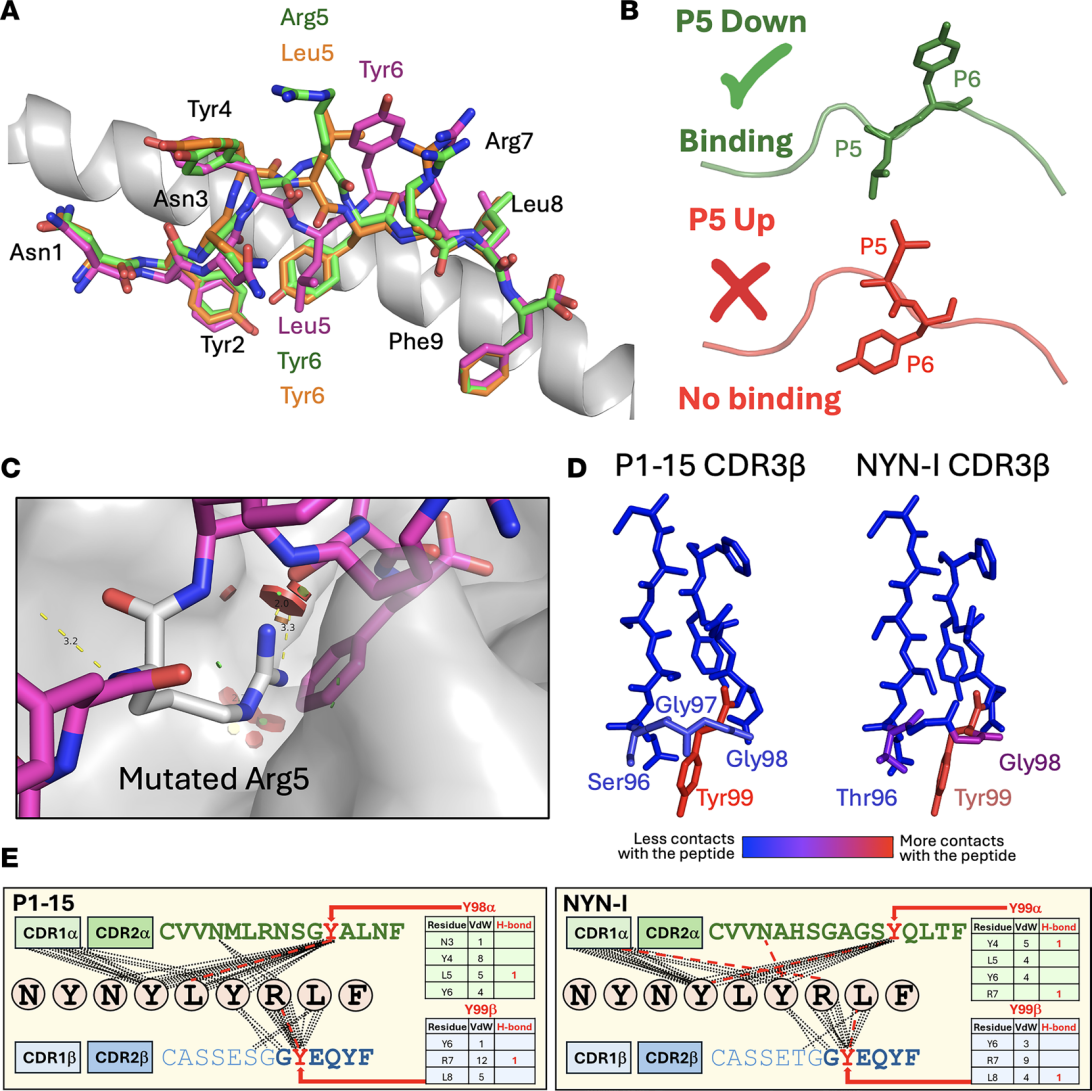
Structural basis for position-5–driven reorientation of the NF9 peptide within HLA-A*24:02. (**A**) A24/NF9 peptide (magenta sticks) in complex with the P1-15 TCR, with uncomplexed A24/NF9-6F (orange sticks) and A24/NF9-5R (green sticks) structures superimposed, demonstrating side chain flipping at positions 5 and 6. (**B**) Diagram illustrating the alternative “P5-up” (top, green) and “P5-down” (bottom, red) peptide orientations adopted within the peptide–HLA complexes in **A**. (**C**) Modeling of the NF9 peptide residue 5 (white sticks) mutated to arginine using PyMOL. Red discs indicate steric clashes; the displayed rotamer produced the fewest clashes after PyMOL energy minimization. (**D**) Structural heatmap showing the number and distribution of atomic contacts between each of the P1-15 and NYN-I TCRs and the NF9 peptide. (**E**) These bonds are also shown as a schematic representation of intermolecular contacts between NF9 peptide residues and the P1-15 (top) or NYN-I (bottom) TCRs, showing how CDR3 tyrosines dominate the molecular contacts with both HLA and peptide. In **D**, blue = no peptide and red = highest contacts with the colors reflecting P1-15 binds much stronger than NYN-1 (K_D_ ≈ 0.67 μM versus > 10 μM).

We next solved the structures of HLA-A*24:02 bound to NF9-6F and NF9-5R at 2.6 Å and 2.3 Å resolution, respectively (Supplemental Figure 7 and Supplemental Table 4). Both variant peptides retained a similar overall conformation to WT NF9 but showed striking rearrangements at P5 and P6 (Figure 6, A and B). In the WT complex, Leu5 pointed downward into the HLA groove, allowing Tyr6 to project upward and form 11 molecular interactions with P1-15. In contrast, the NF9-6F and NF9-5R peptides rotated residue 5 upward, forcing Tyr6 into the groove where it was buried and unavailable for TCR contact. This “P5-up, P6-down” orientation was consistent across all molecules in the crystallographic unit cell, suggesting it represents a stable alternate conformation favored by the escape variants. The previously published A24/NF9 structure (40) contains both conformations within the same unit cell, confirming that “P5-down, P6-up” and “P5-up, P6-down” orientations can coexist in the absence of TCR (Supplemental Figure 7). Additionally, modeling an Arginine residue into P5 of the “P5-down” configuration shows substantial steric hindrance even in the model’s most energetically favorable position (Figure 6C). Together, these observations indicate that the TCR selectively engages the “P5-down, P6-up” configuration, which is disfavored by L452R and related mutations. Contact heatmaps (Figure 6D) and schematic interaction maps (Figure 6E) reveal a tyrosine-centered binding chemistry; CDR3β Tyr99 and CDR3α tyrosines account for most hydrogen-bond and van der Waals contacts to both peptide and HLA helices. This convergence provides a clear structural explanation for the recurrent C-terminal GY(E/Q)QYF motif across A24/NF9-specific clonotypes. Thus, mutation at P5 enforces a conformational reorientation of the NF9 peptide that prevents productive TCR engagement. This conformational reorientation provides a clear structural mechanism for the universal loss of A24/NF9 recognition observed across public TCRs. The L452R mutation thereby reconfigures peptide presentation to prevent engagement by the dominant TCR repertoire.

### HLA-A*24:02 NF9 5L and 5R peptides are not recognized by T cells in Delta-infected convalescents.

**Figure 7:**
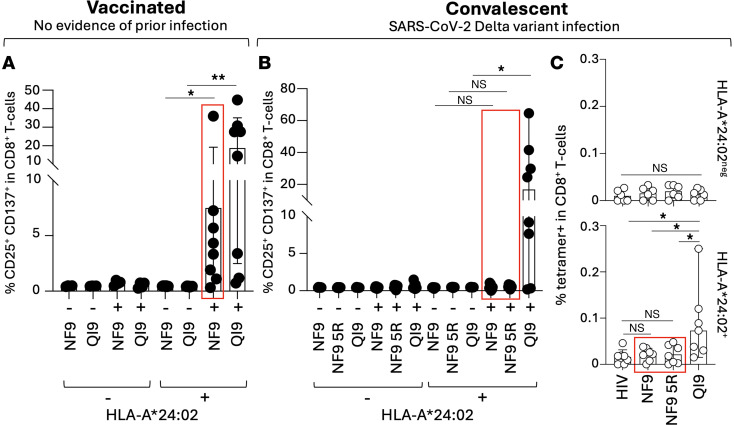
NF9-5R–specific T cells are not detectable in SARS-CoV-2 Delta-infected convalescents. (**A**) Activation-induced marker assay of PBMCs from vaccinated donors with no evidence of prior SARS-CoV-2 infection. Cells were stimulated for 14 days with A24/NF9 (NYNYLYRLF_448–456_) or A24/QI9 (QYIKWPWYI_1208–1216_) peptides. Data show the percentage of CD25^+^CD137^+^CD8^+^ T cells in HLA-A*24:02–negative (*n* = 4) and HLA-A*24:02^+^ (*n* = 8) donors. Wilcoxon matched-pairs signed-rank test (*P = 0.0156, **P = 0.0078). (**B**) Activation-induced marker assay of PBMCs from unvaccinated convalescents infected with the SARS-CoV-2 Delta variant. Cells were stimulated with A24/NF9, A24/NF9-5R, or A24/QI9 peptides. Data show the percentage of CD25^+^CD137^+^CD8^+^ T cells in HLA-A*24:02–negative (*n* = 9) and A*24:02^+^ (*n* = 8) donors. Wilcoxon matched-pairs signed-rank test (*P = 0.0391). (**C**) Ex vivo detection of HLA-A*24:02–restricted tetramer^+^ CD8^+^ T cells in Delta-infected convalescents. PBMCs from HLA-A*24:02–negative (*n* = 6) and A*24:02^+^ (*n* = 7) donors were stained with tetramers specific for HIV-KW9 (negative control), A24/NF9, A24/NF9-5R, or A24/QI9. Wilcoxon matched-pairs signed-rank test (*P = 0.0156 versus A24/HIV-KW9 and A24/NF9-5R and *P = 0.0312 versus A24/NF9).

Viral escape mutations can, in some cases, elicit new variant-specific T cell responses capable of recognizing the altered sequence, as observed in HIV-1 and other rapidly evolving viruses (41). We therefore asked whether individuals infected with the SARS-CoV-2 Delta variant, which harbors the L452R substitution at P5 of the A24/NF9 epitope, could mount new NF9-5R–specific responses. To test this, we analyzed unvaccinated convalescents known to have been infected with the Delta variant and with no prior history of SARS-CoV-2 exposure, representing likely first antigen encounters (Supplemental Table 2). PBMCs were stimulated in vitro with either A24/NF9 or A24/NF9-5R peptides, and proliferating CD8^+^ T cells were assessed by coexpression of CD25 and CD137. In vaccinated HLA-A*24:02^+^ donors, both NF9- and QI9-specific T cells were readily induced (*P = 0.0156 and **P = 0.0078, respectively) (Figure 7A and Supplemental Figure 8). In contrast, PBMCs from HLA-A*24:02^+^ Delta-infected convalescents showed no detectable activation following stimulation with either NF9 or NF9-5R peptides (no significance) but could mount a response to A24/QI9 (*P = 0.0391) (Figure 7B). Ex vivo tetramer staining confirmed the absence of NF9- or NF9-5R–specific CD8^+^ T cells in these donors, whereas robust A24/QI9-specific populations were detected (*P = 0.0316 versus A24/NF9-specific ones and *P = 0.0156 versus A24/NF9-5R–specific ones) (Figure 7C). Together, these data indicate that the L452R substitution abolishes recognition of the A24/NF9 epitope by preexisting vaccine- or infection-induced T cells and simultaneously fails to prime de novo variant-specific responses. Consequently, the Delta variant created a population-level T cell “blind spot” within an otherwise immunodominant region, providing a mechanistic link between structural peptide reorientation and immune escape in HLA-A*24:02^+^ individuals.

## Discussion

In this study, we dissected the molecular basis of the immunodominant HLA-A*24:02–restricted T cell response against SARS-CoV-2 and its failure against emerging variants. By combining population-level analysis of vaccinated and convalescent donors with molecular and structural studies, we demonstrate that a single amino acid substitution at P5 (L452R) within the spike-derived NF9 epitope reorients the peptide within HLA-A*24:02, disrupting TCR engagement and producing complete functional escape. Our data confirm that NF9 represents the dominant HLA-A*24:02–restricted spike epitope after mRNA vaccination and natural infection. The prevalence of shared, “public” TCR clonotypes across unrelated donors, defined by TRAV12-1 and a conserved CDR3β (CASSXXXGYEQYF) motif, highlights the strong structural constraints that underlie this immunodominance. Although such convergence is typically associated with robust antiviral protection, it also creates population-wide vulnerability when a single mutation can abrogate recognition across the entire public TCR repertoire. Using TCR functional assays and crystallography, we show that the NF9 peptide adopts a “P5-down, P6-up” orientation within HLA-A*24:02 that allows the P6 tyrosine to form the dominant TCR contact. The L452R substitution at P5 cannot be accommodated in this conformation and instead forces the peptide into an alternative “P5-up, P6-down” orientation, burying Tyr6 within the HLA groove. This conformational switch eliminates the major TCR binding hotspot, providing a direct structural explanation for universal escape from A24/NF9-specific T cells.

Importantly, Delta-infected convalescents failed to generate new T cell responses against the variant peptide, indicating that L452R creates a T cell “blind spot” rather than simply shifting the epitope specificity. This distinguishes T cell escape in SARS-CoV-2 from that generally observed in HIV-1, where escape can drive secondary variant-specific responses (42, 43). The absence of detectable NF9- or NF9-5R–specific responses in Delta convalescents, despite preservation of other HLA-A*24:02–restricted epitopes such as QI9, demonstrates that mutation at L452 renders this immunodominant site invisible to the HLA-A*24:02–restricted CTL repertoire. It is important to note that loss of an immunodominant CD8 T cell response is unlikely to equate to complete loss of antiviral immunity at the individual level. In principle, removal of a dominant epitope could permit the expansion of previously subdominant T cell responses targeting other viral determinants. However, the A24/NF9 response is exceptional in its magnitude, public TCR architecture, and population prevalence, and we did not observe the emergence of variant-specific or compensatory CD8 T cell responses in Delta-infected convalescents. Moreover, widespread vaccination has artificially skewed human T cell immunity toward the spike protein, further amplifying the dominance of epitopes such as NF9. In this context, escape at NF9 is unlikely to be readily offset by redistribution of immunodominance and, instead, may create a genuine population-level blind spot. By contrast, the more recent L452W substitution, which became widespread during successive SARS-CoV-2 waves and remains common in many Omicron-derived lineages, mediates a similar but incomplete attenuation of T cell recognition. In our dataset, a minority of TCRs displayed weak residual activity toward NF9-5W, whereas none recognized NF9-5R (Figure 3). The ability of NF9-5W to evade most NF9-specific TCRs likely conferred a selective advantage, but its spread within those lineages may also reflect other fitness gains acquired within the parent viral lineage. Our demonstration that some TCRs retain low-level reactivity toward NF9-5W indicates that this variant does not represent true “fixation escape” at the population level. Rather, it may represent a form of incomplete immune escape, in which partial preservation of TCR recognition allows sufficient TCR-recognition to avoid strong purifying selection while maintaining overall viral fitness.

Our results extend earlier observations of L452-mediated escape (13, 14, 34, 44) by defining the structural mechanism that underlies this phenomenon across multiple naturally occurring substitutions at P5. Whereas Tian et al. (45) analyzed T cell reactivity in convalescents infected with BA.2.86 or JN.1, our study combines vaccinated and convalescent cohorts across Wuhan, Alpha, and Delta infection, quantifies the immunodominance of A24/NF9 in human immunity, reconstructs 13 distinct public and private TCRs, and resolves the peptide-HLA rearrangement responsible for universal TCR escape. Our work therefore establishes a mechanistic explanation for the widespread loss of recognition observed in later variants.

Together, these data illustrate a unifying principle: mutations at P5 of A24/NF9 act through conformational reorientation rather than simple disruption of direct contacts. Moreover, the emergence and persistence of L452 substitutions support the broader concept that CD8^+^ T cell pressure can drive viral evolution at immunodominant epitopes once population immunity is established. This process was first documented for HLA-A*02:01–restricted responses in SARS-CoV-2 (9), and the spread of L452 mutations at the dominant A24-restricted epitope now provides parallel evidence in a distinct HLA context. Together, these examples demonstrate that CD8^+^ T cell immunity leaves discernible and predictable signatures on the SARS-CoV-2 genome, consistent with patterns previously observed in HIV-1 and influenza (18–22).

The concept that a single “side chain flip” can destroy a major antiviral response has broad implications for T cell immunology. Although similar conformational “molecular switches” have been described for immunodominant epitopes HIV-1 (46) and melanoma antigens (47), the A24/NF9 system provides the clearest example of this mechanism driving population-level escape in a globally circulating human pathogen. Given the high prevalence of the HLA-A*24:02 allele, particularly across East and Southeast Asia, structural flipping at this epitope offers a compelling explanation for the rapid expansion of variants carrying substitutions at residue 452. Understanding how peptide structural constraints shape T cell vulnerability will be critical for the rational design of next-generation vaccines. In summary, we show that P5-driven reorientation of the immunodominant A24/NF9 epitope underlies universal loss of recognition by public TCRs. This mechanistic insight provides a framework for anticipating viral evolution at structurally constrained epitopes and for prioritizing epitopes that are less amenable to conformational escape as the basis for broadly protective T cell vaccines.

## Methods

### Sex as a biological variable.

Sex was not considered as a biological variable in this study.

### Collection of human PBMCs.

PBMCs were obtained from 28 HLA-A*24:02^+^ donors vaccinated with BNT162b2- or mRNA-1273 (mean age: 38.0, range: 22–67, 71.4%, 20 males and 8 females), 17 HLA-A*24:02^–^ BNT162b2-vaccinated donors (mean age: 34.0, range: 23–57, 12 males and 5 females), 3 HLA-A*24:02^+^ unvaccinated donors (mean age: 39.0, range: 22–56, all males), 12 HLA-A*24:02^+^ convalescents (mean age: 38.0, range: 23–61, 6 males and 6 females), and 9 HLA-A*24:02^–^ convalescents (mean age: 47.0, range: 33–71, 7 males and 2 females). PBMCs were isolated by a density gradient centrifugation using Ficoll-Paque Plus (GE Healthcare Life Sciences, 17-1440-03) and cryopreserved until further use.

### Cell culture.

A549 cells stably expressing human ACE2 and HLA-A*24:02–IRES-GFP (generated previously; ref. 14) were maintained in Ham’s-F12 (Wako, 080-08565) supplemented with 10% FBS. C1R cells expressing HLA-A*24:02 (C1R-A2402) were cultured in RPMI 1640 medium (Thermo Fisher Scientific, 11875101) containing 10% FBS.

### ELISpot assay.

Ex vivo IFN-γ ELISpot assay was performed using anti-human IFN-γ mAbs 1-D1K (Mabtech, Code: 3420-3-1000) and 7-B6-1 (biotinylated detection Mabtech, Code: 3420-6-250) followed by Streptavidin-ALP (Mabtech, Code: 3310-8-1000) and AP Conjugated Substrate Kit (Bio-Rad, 1706432) MultiScreen 96-well plates were prewashed with PBS and blocked with RPMI 1640 medium (Thermo Fisher Scientific, 11875101) containing 10% FBS. Defrosted PBMCs (1 × 10^5^ per well) were stimulated with OLPs (11 aa overlap) spanning the SARS-CoV-2 spike protein (2 μg/mL) for 20 h. Spots were counted using ImmunoSpot (Cellular Technology Limited). Positive responses were defined as >3 spots when the negative control showed 0, or >2× the negative control when background spots were present.

### Tetramer staining.

NF9, NF9-5R, or QI9 and NF9-5R peptide-HLA-A*24:02 tetramers (PE or BV421) were generated using QuickSwitch Quant HLA-A*24:02 Tetramer Kit (MBL International, TB-7302-K1 or TB-7302-K4). PBMCs were stained with tetramers for 30 min at room temperature, followed by surface staining with antibodies to: CD8 (APCcy7 HIT8a, 1/100 dilution; BioLegend), CD14 (PerCP/Cy5.5, HCD14, 1/100 dilution; BioLegend), CD3 (AF532, UCHT1, 1/25 dilution; eBioscience), CD19 (SB436, HIB19, 1/50 dilution; eBioscience). Dead cells were excluded using 7-aminoactinomycin D (BioLegend, 420404). Fixed samples (1% paraformaldehyde, Nacalai Tesque, 09154-85), were acquired on a FACS Canto II (BD Biosciences) or Cytek Northern Lights (Cytek, Japan) and analyzed using FlowJo v10 software.

### Activation induced marker assay.

Activation induced marker assay was performed as previously described (13, 14, 44). Briefly, PBMCs were pulsed with 100 nM NF9, NF9-5R or QI9 peptides (Scrum Inc.) and cultured for 10–14 days. in RPMI 1640 medium with 10% FBS and 30 U/mL IL-2 (PeproTech). Expanded CD8^+^ T cells were restimulated with or without the peptide for 24 h at 37°C and stained with antibodies to CD3 (FITC, UCHT1, 1/100 dilution), CD8 (APCcy7, RPA-T8, 1/100 dilution), CD14 (PerCP/Cy5.5, HCD14, 1/100 dilution), CD19 (PerCP, Cy5.5 HIB19, 1/100 dilution), CD25 (PEcy7, M-A251, 1/50 dilution) and CD137 (APC, 4B4-1, 1/50 dilution; BioLegend). Samples were analyzed by flow cytometry as above.

### The peptide-dependent stabilization assay.

Performed as described (48). TAP-deficient C1R-A24 cells were incubated at 26°C overnight with 1 μM β2-microglobulin (β2m) and graded peptide concentrations in 96-well U-bottom plates. Cells were stained with FITC-labeled Bw4-specific mAb 17A12 (provided by Ulrich Hämmerling; Memorial Sloan Kettering Cancer Center, New York, New York, USA) and analyzed by FACScan (BD, San Jose, CA, USA). Binding was normalized using high-binder peptide (TYLPTNASL) and low-binder peptide (RVWESATPL) controls.

### TCR cDNA amplification from single T cells and construction of TCR expression vector.

A24/NF9^+^ or A24/QI9/A24^+^CD8^+^7-AAD^–^ T cells were single-cell sorted into 96-well plates. TCRα and TCRβ cDNA were amplified using one-step multiplex RT-PCR (31) and sequenced. Clonotypes were analyzed with IMGT/V-QUEST. Assembled TCRβ-P2A-TCRα-P2A-BlaR fragments were cloned into the PiggyBac vector (SBI, PB530A-2) by the Gibson assembly method.

### TCR sensitivity assay.

PB TCR-P2A-BlaR was electroporated into JurkatΔ-Luc with transposase (SBI, PB200PA-1) using Neon (Thermo Fisher Scientific 1200v, 5 ms, 5 pulses). Transfectants were selected with 10 μg/mL of blasticidin-S for 10-14 days. TCR-expressing Jurkat cells were cocultured with A549-ACE2-A2402 cells (E:T ratio of 2:1) for 6 h. Luciferase activity was measured using Steady-Glo (Promega,E2510) on a CentroXS3 plate reader (Berthold Technologies).

### Protein expression and purification.

Soluble P1-15 TCR protein and biotinylated pMHCI were manufactured as previously described (49). Briefly, codon-optimized P1-15 TCRα and TCRβ chains, HLA-A*24:02 heavy chain, and β2m chain gene fragments were generated by GeneArt. All sequences were confirmed by automated DNA sequencing (Eurofins). P1-15 TCR expression constructs were designed with a disulfide-linked construct to produce the soluble domains (variable and constant) for both the α (residues 1–204) and β chains (residues 1–245) (49). The HLA-A*24:02 heavy chain (residues 1–248) (α1, α2, and α3 domains), tagged or not tagged with a biotinylation sequence, and β2m (residues 1–100) were also cloned and used to make the pMHCI complexes. The P1-15 TCRα and TCRβ chains, the HLA-A*24:02 heavy chain, and β2m sequences were inserted into separate pGMT7 expression plasmids under the control of the T7 promoter (50). Competent Rosetta DE3 E. coli cells were used to produce the P1-15 TCRα and TCRβ chains, HLA-A*24:02 heavy chain and B2m in the form of inclusion bodies (IBs) using 0.5 mM IPTG to induce expression and protein were chemically refolded as described previously (51).

### SPR experiments.

TCR:pMHC binding kinetics was determined by SPR as described (52). SPR experiments were conducted using a BIAcore T200 (Cytiva). Biotinylated pMHC molecules were immobilized onto a CM5 sensor chip (Cytiva). HLA-A*24:02–AYAQKIFKIL was bound to flow cell 1 as a negative control, with samples bound to flow cells 2-4. Equilibrium binding analysis was performed at 25°C. Ten serial dilutions of the TCR were made, and 100 mL of each dilution was injected onto the chip. Data were analyzed using GraphPad Prism and fitted to a global fit algorithm. KD values were calculated assuming 1:1 binding using a non-linear fit curve (y = [P1 x]/[P2 + x]).

### Crystallization, diffraction data collection, and model refinement.

TCR and pMHC protein crystals were grown at 18°C by sitting drop vapor diffusion. TCR or peptide-MHC at a concentration of 10 mg/mL in a buffer of 10 mM Tris and 10 mM sodium chloride buffer, was dispensed in 200 nL drops into 3-well, low-profile Intelliplates (Art Robbins Instruments) using the Gryphon liquid handling robot (Art Robbins Instruments). Potential crystallization conditions were created by supplementing the protein with 200 nL from a 96-well crystallization screen and loading the reservoir with 60 mL of the same condition. For the crystallization of TCR/pMHC complexes, both components were mixed in a 1:1 molar ratio and followed the same process as described above. The MIDAS screen (Molecular Dimensions) (53) and the T cell optimized crystal screen were used for this study (54) with the conditions for each structure reported in the relevant statistics tables (Supplemental Table 4).

Crystals were sent to the Diamond Light Source synchrotron in Oxfordshire, UK. X-ray datasets were collected using a PILATUS 9M pixel detector at a wavelength of 0.98 A˚ and consisted of 3,600 images, with 0.1° oscillation and 0.1-second exposure at the I04 MX beamline at the Diamond Light Source. Datasets were processed using the DIALS and AIMLESS pipelines. The CCP4 version 8 software suit (Collaborative Computational Project No. 4) was used to derive 3D models from the reflection intensities (55). Phaser version 2.7 was used to conduct molecular replacement (56), Win-Coot version 0.9.6 was used to match the model to the electron density map and add relevant solvents to the 3D structure (57), and REFMAC version 5.8 was used to refine the 3D structures (58). Three-dimensional protein structures were analyzed, and images were prepared using Pymol version 2.3.4 (Schrodinger LLC). The reflection data and final coordinates were deposited in the PDB database www.rcsb.org (P1-15:HLA-A*24:02-NF9 PDB: 28IL, HLA-A*24:02–NF9-6F PDB: 8RJH, HLA-A*24:02–NF9-5R PDB: 8RJI).

### Statistics.

Data and statistical analysis were performed using Prism 10 (GraphPad Software). For 2-way comparison, 2-tailed Pearson test (Supplemental Figure 1E), unpaired Mann-Whitney *U* test (Figure 1, D and E), or the paired Wilcoxon signed-rank test (Figure 7, A–C) was used.. All experiments were independently replicated at least twice unless otherwise stated.

### Data availability.

All data supporting the findings of this study are available within the manuscript and its supplemental information. Supporting data values for all figures are provided in the Supporting Data Values file. TCR sequences identified in this study are reported in the manuscript and Supplemental Figures 2 and 3. Additional data are available from the corresponding authors upon reasonable request.

### Study approval.

All protocols involving patients at Kumamoto University, Kyushu Medical Center, and Kyushu University were reviewed and approved by the IRB of Kumamoto University (approval nos. 461 and 477). Written informed consent was obtained from all participants.

## Author contributions

TN, AW, GD, LRT, HT, HH, YA, TST, TMT, MT, YG, HL, KU, PJR, and CM performed the experiments. YM, HO, KN, YN, RM, and HN collected clinical samples. MK, HK, and TU prepared reagents. AW and PJR performed crystal structure analysis. AKS and CM designed the experiments and interpreted the results. CM wrote the original manuscript. All authors reviewed and proofread the manuscript.

## Conflict of interest

The authors have declared that no conflict of interest exists.

## Funding support

- AMED Research Program on Emerging and Reemerging Infectious Diseases (20fk0108539h0001 and 20fk0108451s0101 to TU)
- AMED Research Program on HIV/AIDS (21fk0410046 to CM)
- AMED Research Program on Interdisciplinary Cutting-edge Research (23wm0325064 to CM)
- AMED Medical Research Support Program (JP256f0137011 to CM)
- AKS is a Wellcome Investigator (220295/Z/20/Z) and this support funded all work at Cardiff University
- JSPS KAKENHI Grant-in-Aid for Scientific Research (B) (19H03703, 22H03119 to TU) and (22H02877 to CM)
- JSPS KAKENHI Fostering Joint International Research (A) (22KK0277 to CM)
- JSPS KAKENHI Bilateral Program (120259937 to CM)
- Mochida Memorial Foundation for Medical and Pharmaceutical Research (to CM)
- Chemo-Sero-Therapeutic Research Institute (to CM)
- JSPS KAKENHI Grant-in-Aid for Scientific Research (C) (22K07089 to MT)
- Takeda Science Foundation (to CM and MT)
- Intramural grant from Kumamoto University COVID-19 Research Projects (AMABIE) (to CM)
- IMAI Memorial Trust for AIDS Research (to MT)
- Shin-Nihon Foundation of Advanced Medical Research (to MT)

## Supplementary Material

Supplemental data

Supporting data values

## Version 1 03/17/2026

In-Press Preview

## Version 2 05/08/2026

Electronic publication

## References

[1] Rydyznski Moderbacher C (2020). Antigen-specific adaptive immunity to SARS-CoV-2 in acute COVID-19 and associations with age and disease severity. Cell.

[2] Tan AT (2021). Early induction of functional SARS-CoV-2–specific T cells associates with rapid viral clearance and mild disease in COVID-19 patients. Cell Rep.

[3] Le Bert N (2021). Highly functional virus-specific cellular immune response in asymptomatic SARS-CoV-2 infection. J Exp Med.

[4] Sekine T (2020). Robust T cell immunity in convalescent individuals with asymptomatic or mild COVID-19. Cell.

[5] Reynolds CJ (2020). Discordant neutralizing antibody and T cell responses in asymptomatic and mild SARS-CoV-2 infection. Sci Immunol.

[6] Mallajosyula V (2021). CD8^+^ T cells specific for conserved coronavirus epitopes correlate with milder disease in COVID-19 patients. Sci Immunol.

[7] Augusto DG (2023). A common allele of HLA is associated with asymptomatic SARS-CoV-2 infection. Nature.

[8] Kawashima Y (2009). Adaptation of HIV-1 to human leukocyte antigen class I. Nature.

[9] Dolton G (2022). Emergence of immune escape at dominant SARS-CoV-2 killer T cell epitope. Cell.

[10] Stanevich OV (2023). SARS-CoV-2 escape from cytotoxic T cells during long-term COVID-19. Nat Commun.

[11] Agerer B (2021). SARS-CoV-2 mutations in MHC-I-restricted epitopes evade CD8^+^ T cell responses. Sci Immunol.

[12] de Silva TI (2021). The impact of viral mutations on recognition by SARS-CoV-2 specific T cells. iScience.

[13] Motozono C (2021). SARS-CoV-2 spike L452R variant evades cellular immunity and increases infectivity. Cell Host Microbe.

[14] Motozono C (2022). The SARS-CoV-2 Omicron BA.1 spike G446S mutation potentiates antiviral T-cell recognition. Nat Commun.

[15] Chen Y (2023). Structural definition of HLA class II-presented SARS-CoV-2 epitopes reveals a mechanism to escape pre-existing CD4^+^ T cell immunity. Cell Rep.

[16] Tye EXC (2022). Mutations in SARS-CoV-2 spike protein impair epitope-specific CD4^+^ T cell recognition. Nat Immunol.

[17] Bertoletti A (2022). SARS-CoV-2–specific T cells in the changing landscape of the COVID-19 pandemic. Immunity.

[18] Matthews PC (2009). HLA footprints on human immunodeficiency virus type 1 are associated with interclade polymorphisms and intraclade phylogenetic clustering. J Virol.

[19] Sun W (2023). Phenotypic signatures of immune selection in HIV-1 reservoir cells. Nature.

[20] Moore CB (2002). Evidence of HIV-1 adaptation to HLA-restricted immune responses at a population level. Science.

[21] Carlson JM (2015). HIV-1 adaptation to HLA: a window into virus-host immune interactions. Trends Microbiol.

[22] Woolthuis RG (2016). Long-term adaptation of the influenza A virus by escaping cytotoxic T-cell recognition. Sci Rep.

[23] Fujii SI (2022). Association of cellular immunity with severity of COVID-19 from the perspective of antigen-specific memory T cell responses and cross-reactivity. Inflamm Regen.

[24] Chen H (2012). TCR clonotypes modulate the protective effect of HLA class I molecules in HIV-1 infection. Nat Immunol.

[25] Motozono C (2014). Molecular basis of a dominant T cell response to an HIV reverse transcriptase 8-mer epitope presented by the protective allele HLA-B*51:01. J Immunol.

[26] Price DA (2009). Public clonotype usage identifies protective Gag-specific CD8+ T cell responses in SIV infection. J Exp Med.

[27] Gonzalez-Galarza FF (2020). Allele frequency net database (AFND) 2020 update: gold-standard data classification, open access genotype data and new query tools. Nucleic Acids Res.

[28] Kuse N (2022). Long-term memory CD8^+^ T cells specific for SARS-CoV-2 in individuals who received the BNT162b2 mRNA vaccine. Nat Commun.

[29] Shimizu K (2021). Identification of TCR repertoires in functionally competent cytotoxic T cells cross-reactive to SARS-CoV-2. Commun Biol.

[30] Dang TTT (2023). Breadth and durability of SARS-CoV-2–specific T cell responses following long-term recovery from COVID-19. Microbiol Spectr.

[31] Hamana H (2016). A novel, rapid and efficient method of cloning functional antigen-specific T-cell receptors from single human and mouse T-cells. Biochem Biophys Res Commun.

[32] Rowntree LC (2021). SARS-CoV-2–specific CD8^+^ T-cell responses and TCR signatures in the context of a prominent HLA-A*24:02 allomorph. Immunol Cell Biol.

[33] Minervina AA (2022). SARS-CoV-2 antigen exposure history shapes phenotypes and specificity of memory CD8^+^ T cells. Nat Immunol.

[34] Tan TS (2022). Dissecting naturally arising amino acid substitutions at position L452 of SARS-CoV-2 spike. J Virol.

[35] Uriu K (2023). Transmissibility, infectivity, and immune evasion of the SARS-CoV-2 BA.2.86 variant. Lancet Infect Dis.

[36] Sidney J (2005). Classification of A1- and A24-supertype molecules by analysis of their MHC-peptide binding repertoires. Immunogenetics.

[37] Koopmans M (2021). SARS-CoV-2 and the human-animal interface: outbreaks on mink farms. Lancet Infect Dis.

[38] Cole DK (2007). Human TCR-binding affinity is governed by MHC class restriction. J Immunol.

[39] Deng S (2024). Structural insights into immune escape at killer T cell epitope by SARS-CoV-2 Spike Y453F variants. J Biol Chem.

[40] Zhang H (2021). Profiling CD8+ T cell epitopes of COVID-19 convalescents reveals reduced cellular immune responses to SARS-CoV-2 variants. Cell Rep.

[41] Goulder PJ, Watkins DI (2004). HIV and SIV CTL escape: implications for vaccine design. Nat Rev Immunol.

[42] Allen TM (2005). De novo generation of escape variant-specific CD8+ T-cell responses following cytotoxic T-lymphocyte escape in chronic human immunodeficiency virus type 1 infection. J Virol.

[43] Sun X (2016). Effects of a single escape mutation on T cell and HIV-1 co-adaptation. Cell Rep.

[44] Kimura I (2022). The SARS-CoV-2 Lambda variant exhibits enhanced infectivity and immune resistance. Cell Rep.

[45] Tian J (2025). T cell immune evasion by SARS-CoV-2 JN.1 escapees targeting two cytotoxic T cell epitope hotspots. Nat Immunol.

[46] Kloverpris HN (2015). A molecular switch in immunodominant HIV-1-specific CD8 T-cell epitopes shapes differential HLA-restricted escape. Retrovirology.

[47] Bianchi V (2016). A molecular switch abrogates glycoprotein 100 (gp100) T-cell receptor (TCR) targeting of a human melanoma antigen. J Biol Chem.

[48] Mashiba T (2007). Identification of CTL epitopes in hepatitis C virus by a genome-wide computational scanning and a rational design of peptide vaccine. Immunogenetics.

[49] Laugel B (2005). Design of soluble recombinant T cell receptors for antigen targeting and T cell inhibition. J Biol Chem.

[50] Garboczi DN (1996). Structure of the complex between human T-cell receptor, viral peptide and HLA-A2. Nature.

[51] Cole DK (2008). T cell receptor engagement of peptide-major histocompatibility complex class I does not modify CD8 binding. Mol Immunol.

[52] Whalley T (2020). GPU-accelerated discovery of pathogen-derived molecular mimics of a T-cell insulin epitope. Front Immunol.

[53] Grimm C (2010). A crystallization screen based on alternative polymeric precipitants. Acta Crystallogr D Biol Crystallogr.

[54] Bulek AM (2012). TCR/pMHC optimized Protein crystallization screen. J Immunol Methods.

[55] Winn MD (2011). Overview of the CCP4 suite and current developments. Acta Crystallogr D Biol Crystallogr.

[56] McCoy AJ (2007). Phaser crystallographic software. J Appl Crystallogr.

[57] Emsley P, Cowtan K (2004). Coot: model-building tools for molecular graphics. Acta Crystallogr D Biol Crystallogr.

[58] Murshudov GN (2011). REFMAC5 for the refinement of macromolecular crystal structures. Acta Crystallogr D Biol Crystallogr.

